# Virtual Simulation Tools for Communication Skills Training in Health Care Professionals: Literature Review

**DOI:** 10.2196/63082

**Published:** 2025-05-06

**Authors:** Manuel Fernández-Alcántara, Silvia Escribano, Rocío Juliá-Sanchis, Ana Castillo-López, Antonio Pérez-Manzano, M Macur, Sedina Kalender-Smajlović, Sofía García-Sanjuán, María José Cabañero-Martínez

**Affiliations:** 1 Department of Health Psychology, Faculty of Health Sciences, University of Alicante, Alicante, Spain; 2 Institute of Health and Biomedical Research of Alicante, Alicante, Spain; ^3^Department of Nursing, Faculty of Health Sciences, University of Alicante, Carretera San Vicente del Raspeig s/n, Alicante, 03690, Spain; 4 University of Murcia, Murcia, Spain; 5 Angela Boškin Faculty of Health Care, Spodnji Plavž 3, Jesenice, Slovenia

**Keywords:** communication skills, virtual patient, virtual simulation, health care professionals, virtual simulation tool, skill training, communication, heterogeneous, heterogeneous characteristics, virtual tool, patient-centered, patient-centered communication, implementation

## Abstract

**Background:**

Quality clinical care is supported by effective patient-centered communication. Health care professionals can improve their communication skills through simulation-based training, but our knowledge about virtual simulation and its effectiveness and use in training health professionals and students is still growing rapidly.

**Objective:**

The objective of this study was to review the current academic literature to identify and evaluate the virtual simulation tools used to train communication skills in health care students and professionals.

**Methods:**

This review was carried out in June 2023 by collecting data from the MEDLINE/PubMed and Web of Science electronic databases. Once applicable studies were identified, we recorded data related to type of technology used, learning objectives, degree of learning autonomy, outcomes, and other details.

**Results:**

We found 35 articles that had developed and/or applied a virtual environment for training communication skills aimed at patients, in which 24 different learning tools were identified. Most had been developed to independently train communication skills in English, either generally or in the specific context of medical history (anamnesis) interviews. Many of these tools used a virtual patient that looked like a person and had the ability to vocally respond. Almost half of the tools analyzed allowed the person being trained to respond orally using natural language. Of note, not all these studies described the technology they had used in detail.

**Conclusions:**

Many different learning tools with very heterogeneous characteristics are being used for the purposes of communication skills training. Continued research will still be required to develop virtual tools that include the most advanced features to achieve high-fidelity simulation training.

## Introduction

Effective patient-centered communication is one of the key components of quality clinical care [1]. Thus, it is vital that health care professionals adequately manage their communication skills. This involves mastering the transmission of information; listening and comprehensively understanding all the issues related to the health of each patient [2]; and responding appropriately to the physical and emotional needs of patients [3]. Better communication when supporting decision-making means that patients are better able to understand their situation, feel better informed, and are more active in the decision-making process [4]. Hence, acquiring good communication skills has been related to improved health outcomes, general patient satisfaction [5], better adherence to treatment plans [6], and positive effects on health care costs and length of hospital stay [7].

However, despite recognizing the importance of communication, health professionals are not always sufficiently skilled in this area [8]. Therefore, it is advisable that both health and educational institutions introduce different means of supporting the development of communication skills into their training plans as a priority objective. Furthermore, this training must also be implemented through effective educational strategies [9]. It has previously been shown that simulation-based learning is an effective means of acquiring communication skills [9]. Specifically, simulation with a standardized or simulated patient, which consists of using trained people to realistically portray a patient within learning contexts [10], is widely used to train communication skills [1].

Nonetheless, although the use of simulation methodologies has greatly advanced training in communication skills, its implementation also has limitations. For example, in terms of the human resources used in this type of training, it is particularly difficult to recruit actors able to simulate patients precisely and consistently in a completely standardized way [11,12]. Other difficulties include temporal–spatial issues because the availability of simulations with standardized patients is limited to a specific physical space and time [13]. A training alternative that could overcome these limitations is the use of standardized virtual patient programs that use computerized characters rather than real actors [14].

Indeed, compared to standardized patients, there are significant advantages to the use of virtual patients, including the need for fewer staff and resources once developed [15], unlimited availability, and the fact that they are highly customizable [14]. Additionally, these tools provide highly interactive, engaging, and more standardized experiences because educators can control their design, programming, delivery, and use [14]. It is also worth noting that these solutions can be personalized according to specific individual needs, given that they are not limited by time or space, so students can repeatedly engage in training in more clinical scenarios than is possible through traditional methods [15]. In addition, this technology also allows students to learn in a safe environment with low levels of risk and anxiety, which encourages them to gain greater personal awareness of their learning processes [16].

Virtual simulation has gained attention in recent years as a promising tool for training both undergraduate and graduate students, as well as health care professionals, in various competencies, including nontechnical skills. This growing interest is evident in an increasing number of studies focused on its potential applications in health care education [17]. However, despite this expanding body of research, it is advisable to continue researching with the aim of fully exploring and understanding which technological and technical skills are more suitable to train in virtual simulation [17]. Some reviews on virtual simulation and the learning of nontechnical skills such as communication are available [17-19]. For example, in their integrative review, Peddle et al [19] examined how interactions with virtual patients impacted nontechnical skills in general, without exclusively focusing on communication skills or technical and instructional design characteristics. Subsequently, both the systematic review by Lee et al [18] and the literature review by Battegazzorre et al [17] examined the technical characteristics of virtual learning applications aimed at improving communication skills. However, it is noteworthy that these reviews include studies published only up to December 2018 and May 2020, respectively, which highlights a gap in the literature regarding recent advancements in virtual simulation technologies.

The development of communication skills is fundamental for the effective clinical practice of health care professionals. However, the increasing diversity of virtual simulation tools and the rapid pace of technological innovation pose significant challenges to understanding which tools are most effective for training these skills. This raises the following key questions: what are the characteristics of the current virtual simulation tools used for training communication skills, and how effective are they in fostering realistic and immersive learning experiences? To address these questions, we conducted a systematic review of the virtual simulation tools available to train communication skills in health care professionals, analyzing their design, degree of immersion, and autonomy to identify their strengths and limitations.

Therefore, the objective of this study was to review the current academic literature to identify and evaluate the virtual simulation tools used to train communication skills in health care students and professionals and to assess their effectiveness and limitations in training health care personnel.

## Methods

### Design

We completed a literature review to identify virtual simulation tools designed to train communication skills in health care professionals, including students in training and practicing professionals. The inclusion criteria were studies that examined (1) virtual simulation tools and/or those based on artificial intelligence (AI), (2) tools used to train communication skills in health professionals, and (3) tools targeting training in communication skills and/or therapeutic relationships with patients. Studies were excluded if (1) the tools were designed to train interprofessional communication, (2) the objective was noneducational, and (3) the tool was designed to train patients in social and/or communication skills. This systematic review was conducted in accordance with the PRISMA (Preferred Reporting Items for Systematic Reviews and Meta-Analyses) 2020 [20] guidelines to ensure comprehensive and transparent reporting of the methodology and findings.

### Search Strategy

The search for studies was conducted in June 2023 in the MEDLINE/PubMed and Web of Science electronic databases. As part of the search strategy, we consulted the PubMed thesaurus using the following Medical Subject Headings (MeSH) terms: “Artificial Intelligence,” “Machine learning,” “virtual reality,” and “social skills.” The natural language search terms included in the title and/or abstract fields were “artificial intelligence,” “machine learning,” “virtual reality,” “e-simulation,” “web-based simulation,” “virtual simulation,” “virtual patient,” “social skills,” “interpersonal skills,” “social ability,” “social competences,” and “communication skills.” The complete search strategy was as follows: (((“Artificial Intelligence”[MeSH Terms] OR “Machine Learning”[MeSH Terms] OR “Artificial Intelligence”[Title/Abstract] OR “Machine Learning”[Title/Abstract])) OR ((“Virtual Reality”[MeSH Terms] OR “Virtual Reality”[Title/Abstract] OR “e-simulation”[Title/Abstract] OR “web-based simulation”[Title/Abstract] OR “virtual simulation”[Title/Abstract]) OR (“virtual patient”[Title/Abstract]))) AND ((“Social Skills”[MeSH Terms] OR “Social Skills”[Title/Abstract] OR “interpersonal skills”[Title/Abstract] OR (“social ability”[Title/Abstract] OR “social abilities”[Title/Abstract]) OR (“social competence”[Title/Abstract] OR “social competences”[Title/Abstract]) OR “communication skills”[Title/Abstract])).

No temporal restrictions were applied in any of these cases. Despite previous reviews focusing on similar topics [17-19], it was decided not to base the current review on them. This decision was due to differences in the search strategy used, which did not account for the wide range of synonyms associated with each term established for this review. Furthermore, it is important to note that Lee et al [18] focused their strategy exclusively on communication among medical students, while Peddle et al [19] directed their attention to all nontechnical skills, not just communication skills.

The eligibility of the studies was independently assessed by 2 of the authors (MJCM and RJS) and any discrepancies were resolved by another author (SE).

### Data Extraction

Data related to the characteristics of the studies (publication year, country, language, objective, and type) as well as data related to the outcome of the use of the digital/virtual training tool for improving communication skills in health care professionals were recorded. Specifically, we noted the tool name, training language, learning objective, degree of learning autonomy (fully autonomous vs instructor-mediated training), patient type (avatar/doll, virtual patient with a human-like appearance, real person, etc), type of answers given by the trainee (written or oral conversation), and type of technology used.

## Results

### Overview

**Figure 1.**
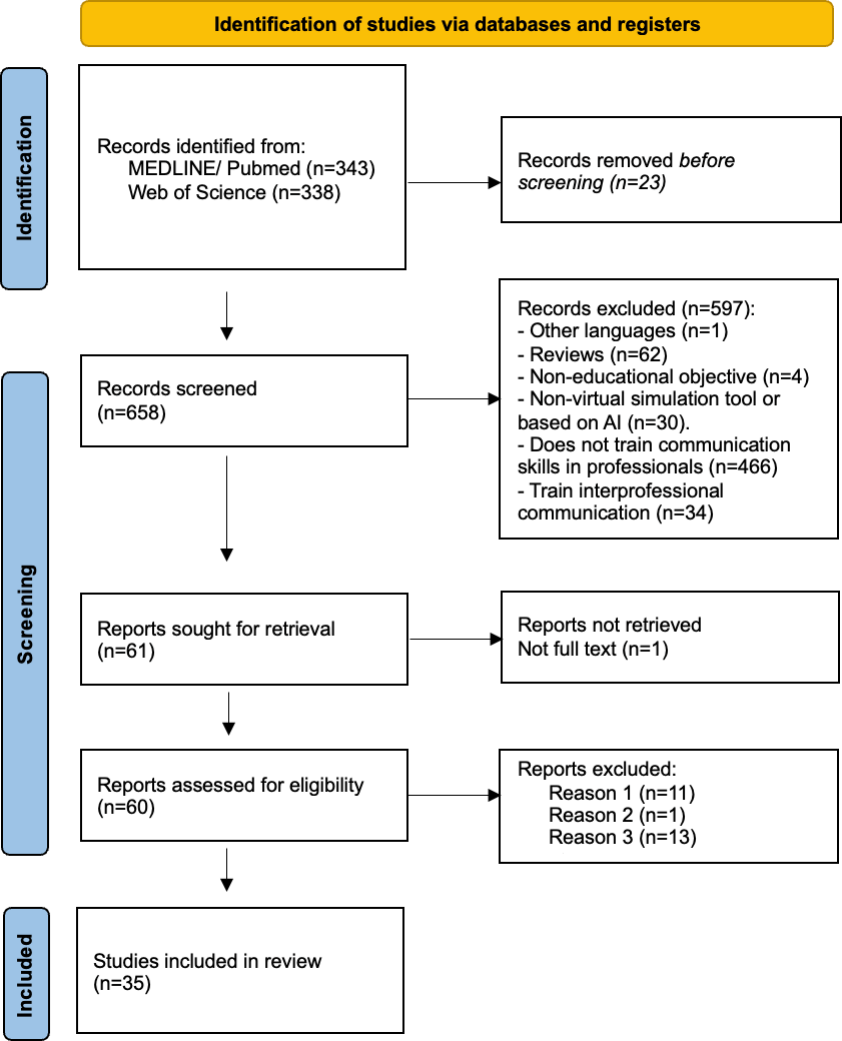
PRISMA flowchart. Reason 1: articles not directly related to training clinical communication skills with patients; reason 2: did not study virtual training; reason 3: did not use a tool designed for training purposes. AI: artificial intelligence; PRISMA: Preferred Reporting Items for Systematic Reviews and Meta-Analyses.

The studies were manually screened and coded. Our search of PubMed and the Web of Science produced 681 records, of which 23 duplicates were eliminated. During the screening process, 2 of the authors independently analyzed 658 studies based on their titles and abstracts (Figure 1). After this initial screening, the full texts of 61 records were obtained for analysis. We requested the full texts of a further 2 articles from the corresponding authors by email and through ResearchGate; of these, we included 1 in this review. Of these 60 studies, 25 were excluded because they did not meet the inclusion criteria. Specifically, 11 articles had not directly trained clinical communication skills with patients (criterion 1), 1 had not studied virtual training (criterion 2), and 13 had not used a tool designed for training purposes (criterion 3). Therefore, a total of 35 articles were included in the review. Finally, one of the authors extracted the relevant data from these 35 studies and entered them into a database following the coding manual we had prepared for this purpose.

### Characteristics of the Studies Included

A total of 35 articles were obtained that had developed and/or applied a virtual environment for training communication skills that would be directed toward patients; overall, 43% (n=15) were articles published in the United States and 17% (n=6) were from Australia, with the remaining articles having been published in Europe and Asia (Table 1). All the articles had been published in English and their objectives are shown in Table 1.

**Table 1.** Description of the studies (N=35).

Articles	Country	Language	Objective
Ali et al [21], 2020	United States	English	Describe the iterative participatory design of SOPHIE, an online virtual patient for “practice” based on feedback from sensitive conversations between patients and clinicians and discuss an initial qualitative evaluation of the system by professional end users.
Bánszki et al [22], 2018	Australia	English	Explore a novice clinical educator’s experience in training essential communication and interpersonal skills using a virtual patient.
Bearman and Cesnik [23], 2001	Australia	English	Assess students’ attitudes toward learning communication skills through a virtual patient; compare the acceptability of the 2 distinct types of virtual patient designs.
Bearman et al [24], 2001	Australia	English	Compare 2 types of virtual patients to understand how different virtual patient designs affect the student learning experience.
Bearman [25], 2003	Australia	English	Explore the students’ experience with the virtual patient.
Borja-Hart et al [26], 2019	United States	English	Assess students’ confidence and impressions when using their communication skills with a virtual patient and evaluate their competencies in the use of this technology.
Chae et al [27], 2023	Korea	English	The purpose of this study was to describe the development of SimCARE and evaluate the feasibility of its use in nursing education.
Courteille et al [28], 2014	Sweden	English	To investigate the dynamics and congruence of interpersonal behaviors and socioemotional interaction exhibited during the learning experience in a virtual patient, and to evaluate which interaction design features contribute most to behavioral and affective engagement in the medical student.
Deladisma et al [29], 2008	United States	English	Develop a virtual training environment system that can be accessed independently.
Dickerson et al [30], 2006	United States	English	Provide information about the advantages and disadvantages of using synthesized speech and evaluate the fidelity necessary for the training of communication skills.
Du et al [31], 2022	China	English	To evaluate the history-taking skills of nursing undergraduates using a virtual standardized patient, and to explore its independent influencing factors.
Guetterman et al [32], 2019	United States	English	To investigate the differential effects of a virtual patient–based simulation developed to train health care professionals in empathetic patient-provider and interprofessional communication.
Hwang et al [33], 2022	Taiwan, Japan	English	A virtual patient–based social learning approach is proposed to enhance nursing students’ performance and clinical judgment in education programs.
Jacklin et al [34], 2018	United Kingdom	English	Create a virtual patient that simulates a primary care consultation, offering the opportunity to practice decision-making. A second objective was to involve patients in the design of a virtual patient simulation and inform the design process.
Jacklin et al [35], 2021	United Kingdom	English	This study aims to evaluate a virtual patient workshop for medical students aimed at developing the communication skills required for shared decision-making.
Kleinsmith et al [2], 2015	United States	English	Develop an understanding of whether students can respond empathically to expressions of concern from a virtual patient.
Lok [36], 2006	United States	English	Teach communication skills using virtual humans.
Maicher et al [37], 2019	United States	English	Describe a virtual standardized patient system that allows students to practice their history-taking skills and receive immediate feedback.
Mayor Silva et al [38], 2023	Spain	English	The objective was to develop a virtual reality simulator to improve communication skills and compare its results with a traditional workshop based on cases and theoretical content explained through video.
Nakagawa et al [39], 2022	Japan	English	The objective structured clinical examination is among validated approaches used to assess clinical competence through structured and practical evaluation.
Ochs et al [40], 2019	France	English	Evaluate the virtual reality training platform in which the user experience is analyzed based on the virtual environment.
Perez et al [41], 2022	United States	English	The purpose of this study was to explore the use of virtual simulation to experience difficult conversations and to evaluate differences in perceptions between nurse educator, family nurse practitioner, and nurse anesthesia students.
Plass et al [42], 2022	Germany	English	The purpose of this study is to evaluate the effectiveness of a brief virtual role-play motivational interviewing training program on motivational interviewing knowledge and skills in first-year undergraduate medical students, making use of both a pre-test and a then-test (retrospective pre-test) to check for response shift in evaluating the educational intervention.
Quail et al [12], 2016	Australia	English	Investigate students’ communication skills, knowledge, confidence, and empathy in simulated and traditional learning environments.
Real et al [43], 2017	United States	English	Develop an immersive virtual reality curriculum on addressing flu vaccine hesitancy using Kern’s 6-step approach to curriculum design. The goal of the program was to teach best communication practices in cases of questions about the flu vaccine.
Real et al [44], 2017	United States	English	Create an immersive virtual reality curriculum to teach pediatric residents communication skills when discussing flu vaccination. Compare effectiveness with a control group.
Real et al [45], 2022	United States	English	Examined the acceptability and tolerability of the approach and the impact of deliberate practice using virtual reality simulations on clinicians’ confidence related to shared decision-making communication skills.
Rouleau et al [46], 2022	Canada	English	This study aimed to assess the acceptability of a virtual patient simulation to improve nurses’ relational skills in a continuing education context.
Sapkaroski et al [47], 2022	Australia	English	The aim of this study was to establish whether the mode of delivery, virtual reality simulated learning environments versus clinical role-play, could have a measurable effect on clinical empathic communication skills for magnetic resonance imaging scenarios.
Sezer and Sezer [48], 2019	Turkey	English	Design, develop, and evaluate a 3D virtual patient application that can move, has voice and lip synchronization, allows written communication, and is supported by a solid scenario to improve students’ communication skills.
Şimşek Çetinkaya et al [49], 2022	Turkey	English	This study aimed to determine the effectiveness of 2 simulation types used for family planning consultation of midwifery students and to compare these methods.
Shorey et al [50], 2019	Singapore	English	Develop and evaluate the use of virtual patients to better prepare undergraduate nursing students to communicate with real-life patients, their families, and other health care professionals during their clinical stays.
Shorey et al [51], 2020	Singapore	English	To examine user attitudes and experiences and clinical facilitators’ perspectives on student performance in the clinical environment following virtual patient training.
Shorey et al [52], 2023	United States	English	This study aimed to evaluate the effectiveness of this theory-based virtual intervention on nursing students’ learning attitudes, communication self-efficacy, and clinical performance.
Stevens et al [53], 2006	United States	English	Create an interactive virtual clinical scenario of a patient with acute abdominal pain to teach medical students history-taking and communication techniques.

### Features of the Virtual Tools

After reading the full text of the 35 articles, we identified 24 different learning tools that had been developed to train communication skills in students or health professionals (Table 2). Most of them (n=15; 62%) had provided training in English [2,21,22,24,26,28,29,32,34,37,41,43,46,47,52]. Regarding the learning objective of the virtual environment, 42% (n=10) aimed to train communication skills in the specific contexts of a clinical history and/or anamnesis interview [2,29,31,33,35,37,42,46,48,52], 42% (n=10) taught general communication skills [22,24,26-28,38,39,41,47,49], and 8% (n=2) covered giving bad news [21,40]. There was also a tool that had been specifically developed to train communication skills to address flu vaccination hesitancy [43-45]. Another tool that had been used to train communication skills focused on empathy is also worth highlighting [32].

**Table 2.** Virtual tools and their characteristics (n=24 tools).

Articles	Tool name	Language	Study purpose	Degree of learning autonomy	Patient type	Type of student responses during training	Type of technology used
Ali et al [21], 2020	SOPHIE	English	Train communication skills for the delivery of bad news. Aimed at health professionals.	Autonomous	Virtual patient with the appearance of a person.Responded with a voice. The entire transcript can be seen.	Oral conversation	Artificial intelligence
Bánszki et al [22], 2018; Quail et al [12], 2016	Not specified	English	Training communication skills. Aimed at health care students.	An instructor mediated the training.	Virtual patient with the appearance of a person. Responded with a voice.	Oral conversation	The instructor was in another room where they controlled everything and responded in the simulated interaction.
Bearman and Cesnik [23], 2001; Bearman et al [24], 2001; Bearman [25], 2003	Not specified	English	Training in communication skills. Aimed at medical students.	Autonomous	Real person speaking. Viewing of recorded videos.	Written. Choice of 3 or 4 written response options available after each video. The authors developed 2 types of responses to compare which was more effective: narrative (detailed communicative structures) or problem-solving (labels with possible actions).	A total of 154 recorded videos. The next video shown was adjusted depending on the response given. Therefore, the virtual patient became satisfied according to responses chosen by the student.
Borja-Hart et al [26], 2019	Used *Shadow Health* from Elsevier	English	Training in communications skills. Aimed at pharmacy students.	Autonomous	Virtual patient with the appearance of a person. Responded with a voice.	Natural language (written and spoken). Students could choose the interaction they would carry out: ask, empathize, or educate.	*Shadow Health* is simulation software that generates different scenarios. The article did not explain any more about the technology used.
Chae et al [27], 2023	SimCARE	Korean	Training in intercultural communication skills. Aimed at nursing students.	Autonomous	Virtual patient with the appearance of a person.Responded with a voice.	They selected a written response from among those on offer.	A virtual reality headset.The authors described the technology used to generate the 3D graphics (Unity 2019.4.0f1 game engine), avatars (DAZ 3D software), and avatar animation (iClone 7).
Courteille et al [28], 2014	Not specified	English and Swedish	Training in communication skills. Aimed at medical students.	Autonomous	Real person speaking. Viewing of recorded videos.	Written. Students replied in text written in natural language.	Interactive Simulation of Patients. A database with 200 videos for each case, allowing the simulator to respond according to the question posed by the student.
Deladisma et al [29], 2008; Dickerson et al [30], 2006; Lok [36], 2006; Stevens et al [53], 2006	Not specified	English	Training in communication skills and anamnesis techniques. Aimed at medical students.	Autonomous but with availability of additional resources. The technology that drives this interaction largely consisted of commodity hardware and software: 2 desktop computers, 2 cameras, a data projector, and a wireless microphone.	Virtual patient with the appearance of a person (an avatar called Diana) who spoke and produced natural gestures. The authors developed 2 types of communication for the avatar to study which one was more effective: real recorded communication or virtual communication.	Oral conversation. The students could speak using natural language. The software also detected various gestures.	The speech recognition worked using *Dragon Naturally Speaking* by Scansoft, which is a database developed with content organized in semantic categories to detect the communicative structures used by the students.
Du et al [31], 2022	University A Virtual Patient (UA-VP, 2021)	Chinese	Training in communication skills to carry out a nursing evaluation by following Gordon’s Functional Patterns.	Autonomous	A virtual patient with the appearance of a person.Responded with text based on a predefined chat.	Written and oral conversation	Recognizes structures and offers feedback based on the uploaded chat scripts (as bullet points and not reflecting the most important part of the interaction). Used WeChat, a social media app.
Guetterman et al [32], 2019	Used MPathic-VR	English	Trained empathic communication skills. Aimed at medical students.	Autonomous	Virtual patient with the appearance of a person.Responded with a voice.	Oral conversation. It also detected gestures and movements.	Artificial intelligence.
Hwang et al [33], 2022	Not specified	Chinese	Trained students in diagnosis and treatment and has a specific medical history module which trains communication skills.	Autonomous	Virtual patient with the appearance of a person.Responded with voice and text.	Did not specify	Learning system designed as a decision tree.
Jacklin et al [34], 2018; Jacklin et al [35], 2021	Not specified	English	Training in communication skills for shared decision-making during clinical interviews. Aimed at medical and/or pharmacy students.	Autonomous	Virtual patient with the appearance of a person.Responded through a voice and with gestures.	Written text. Choice of 3 answer options.	A web-based virtual patient simulator.
Kleinsmith et al [2], 2015	Neurological Examination Rehearsal Virtual Environment	English	Trained communication skills for use during clinical interviews. Aimed at nursing students.	Autonomous	Virtual patient with the appearance of a person.A virtual patient responded with a voice and through text.	Written. The student inserted text written in natural language.	Virtual People Factory.A database used by the simulator to respond based on the student’s question.
Maicher et al [37], 2019	Not specified	English	Trained skills for performing an anamnesis (to collect medical information). It does not address communicative listening strategies such as empathy. Aimed at medical students.	Autonomous	Virtual patient with the appearance of a person. Responded with voice and text.	Oral conversation. Text could also be written.	Artificial intelligence.The open-source natural language processing engine ChatScript is used for the conversion element.Unity gaming platform.
Mayor Silva et al [38], 2023	Not specified	Spanish	Training in communication skills. Aimed at nursing students.	An instructor mediated the evaluation.	Not specified	Not specified	A virtual reality headset.
Nakagawa et al [39], 2022	Not specified	Japanese	Trained communication skills such as desire suppression, expectation acceptance, facial expression, emotional communication, dominance, maintaining relationships, and dealing with disagreements. Aimed at pharmacy students.	Autonomous	A chatbot. Written and oral.	Oral conversation in natural language	Artificial intelligence.If the artificial intelligence did not detect the keywords, the conversation did not continue.There was no direct feedback.
Ochs et al [40], 2019	ACORFORMed	French	Training in the delivery of bad news. Aimed at medical practitioners (students and professionals).	Autonomous in some functions (eg, dialogue generator). In others (eg, categorizing the response and sending it to the simulator), the instructor mediated the learning.	Virtual patient with the appearance of a person.Responded with a voice.	Oral conversation	A virtual reality headset.The instructor categorized the response using a previously coded database and sent that information to the simulator.
Perez et al [41], 2022	Used the Mursion tool	English	Trained communication skills for use in difficult conversations. Aimed at nursing students.	Autonomous	Virtual patient with the appearance of a person.Responded with a voice.	Oral conversation in natural language.	Artificial intelligence (using the Mursion tool).
Plass et al [42], 2022	Used the Kognito Conversarion Platform	German	Training in person-centered communication skills for motivational interviewing. Aimed at medical students.	Autonomous	Virtual patient with the appearance of a person.Responded with a voice.	Select between different answer options.	Artificial intelligence (using the Kognito Conversation Platform).
Real et al [43], 2017; Real et al [45], 2022; Real et al [44], 2017	Not specified	English	Training in communication skills to inform patients about vaccination. Aimed at medical residents.	An instructor mediated the training.	Virtual patient with the appearance of a person.Responded through a voice and with gestures.	Oral conversation and natural language.	Unity gaming platform.A virtual reality headset.
Rouleau et al [46], 2022	Not specified	French, English	Training in nursing relational skills for use in motivational interviews.	Autonomous	Virtual patient with the appearance of a person.Responded with a voice.	Select between different answer options	Used the MedicActiV platform
Sapkaroski et al [47], 2022	Not specified	English	Training in communication skills. Aimed at medical students.	Autonomous	Virtual patient with the appearance of a person.Responded with voice and text.	Select from among answer options. This part of the case simulation was mandatory. It was also capable of natural language oral conversation and the ability to ask alternative questions was optional.	Clinical Education Training Solution virtual reality clinic software using the Oculus Rift CV1 virtual reality headset.
Sezer and Sezer [48], 2019	Not specified	Turkish	Training in basic communication skills for use in a medical interview. Aimed at training health care students.	Autonomous	Virtual patient with the appearance of a person.Responded with a voice and in writing.	Natural written text	Virtual People Factory for avatar and simulation generation. The scenario was created in Unity 3DTM. Different variations of the simulation interventions the students could apply at each stage were included and these answer combinations were compared to the closest preprogrammed scenario to give an answer.
Şimşek Çetinkaya et al [49], 2022	Not specified	Turkish	Training in communication skills for use in a family planning consultation. Aimed at midwifery students.	The instructor offered feedback after watching the simulation.	The patient type was not specified. Responded with a voice.	Oral conversation	Not specified
Shorey et al [50], 2019; Shorey et al [51], 2020; Shorey et al [52], 2023	Virtual Counselling Application using Artificial Intelligence	English	Trained basic communication skills for use in an interview. Aimed at nursing students.	Autonomous	Virtual patient with the appearance of a person.Responded with a voice and in writing.	Oral conversation in natural language	Artificial intelligence.Used the Dialogflow chatbot from Google Cloud to store and process natural language. The scenario was created in Unity 3D.

Several major virtual tools were identified in this review for training communication skills in health care professionals. SOPHIE [21] is a tool designed to train the delivery of bad news using a virtual patient that interacts through oral conversations, leveraging AI. Shadow Health [26] focuses on communication skills for pharmacy students, allowing both written and spoken interactions with a virtual patient. SimCARE [27] is a virtual reality–based tool aimed at nursing students, training intercultural communication skills through animated avatars. MPathic-VR [32] trains medical students in empathic communication, featuring virtual patients that respond with voice and detect nonverbal cues like gestures. ACORFORMed [40] trains medical practitioners in delivering bad news through virtual reality interactions with a virtual patient. Mursion [41] is designed for nursing students to practice difficult conversations using natural language processing for realistic interactions, while the Kognito Conversation Platform [42] supports motivational interviewing through person-centered communication training with virtual patients. VCAAI [50-52] trains basic communication skills in nursing interviews. These tools highlight the diversity of approaches in the use of virtual patients for communication training. Finally, 14 virtual tools did not specify their name.

Some (n=19, 79%) of the tools allowed students to train completely autonomously, whereas 21% (n=5) required an online instructor to mediate the training and respond during the interactions [22,39,40,44,49]. One of the tools could be defined as partially autonomous because a trained instructor had to perform some of the functions [40]. Regarding the patient type used for the training, the vast majority of the tools used virtual patients (n=19; 79%) with the appearance of a real person [2,21,22,26,29,31-33,35,37,40-42,44,46-48,51]. Of these, 95% (18/19) responded with a voice (18/24, 75%), except for the tool published by Du et al [46]. Two tools (8%) used videos recorded with real people [24,28].

Regarding the types of responses the user could give during the training, almost half of the tools analyzed (n=11, 45%) allowed the user to respond orally using natural language [21,22,26,29,31,32,37,39,41,44,49,51]. Shadow Health [26], for example, offers both written and spoken interactions, while SOPHIE [21] focuses solely on oral communication.

## Discussion

This study reviews and analyzes the 24 virtual simulation tools available for training communication skills in health care professionals, assessing their characteristics, levels of immersion, and the autonomy they provide in learning processes. Although virtual simulation tools have shown significant growth in recent years, driven by technological advances, the review identified a high degree of heterogeneity in the approaches, technologies, and interaction methods used. This variety has made it challenging to standardize and effectively integrate these tools into consistent training plans. Most tools rely on virtual patients with a limited range of interaction capabilities, and very few offer fully immersive experiences that mimic real-world clinical communication. Furthermore, limited accessibility to tools in languages other than English, as well as a lack of high-fidelity technologies for simulating realistic, natural language–based conversations, continue to pose significant challenges. Considering these challenges, this review highlights several key findings regarding the applications of virtual environments to enhance communication skills training that will be detailed in the following paragraphs.

First, it is important to highlight the large number of different applications we identified that have been used to improve communication skills (either in basic or more specific situations) through virtual environments. Similarly, other reviews have also concluded that the use of virtual patients for clinical communication training has grown exponentially over the last decade [17,18], which has been driven by rapid technological advances [54], also providing further evidence of the benefits associated with this type of resource [18]. In fact, this work has included 13 new virtual simulation environments developed based on the published review by Battegazzorre et al [17].

Most of the applications we considered in this review used English, which could represent an obstacle for professionals and students who do not know this language. Indeed, only one of the tools identified used Spanish and in this case, it was also mediated by an instructor, thereby making it difficult for students to use it autonomously and independently [38] Therefore, there is still a long way to go to make these tools highly accessible at an international level. Regarding the more technical characteristics, we observed visible heterogeneity in the types of technologies used, including in the different types of patients used for training—for example, the use of chatbots, images, and/or recordings of real people and virtual patients. However, our results showed that almost all the applications we identified had designed virtual environments using virtual patients that looked like a person and could vocally respond to and receive oral responses to simulate a real conversation [21,22,26,29,32,37,39,41,44,49,51]. A key implementation across the tools was the use of natural language processing to simulate realistic conversations.

Training in simulation environments that assume an appropriate level of fidelity (a 3D term that includes physical/environmental, psychological, and conceptual elements) increases realism [55] and influences learning engagement [56]. For example, in their systematic review, Kaplonyi et al [1] reflected how simulations with the use of standardized patients are considered realistic environments and an effective means for learning communication skills. Indeed, the academic literature proposes that virtual patients can be used as a complementary alternative to working with standardized patients [57] and can represent patients in a realistic clinical environment [17] to effectively help students to acquire or improve their communication skills [18]. Nonetheless, it will be important for future lines of research to use standardized tests to evaluate the beneficial effects of training with this type of virtual tool before fully integrating them into training plans [18,54].

In terms of the fidelity of these tools, increasing the immersion of virtual simulations—defined as the psychological state of the perception of being inside or surrounded by something [58]—by using virtual patients with natural language processing and auditory and visual behavior [17,59] is positively related to better communication skills performance [17,19]. However, we must not forget that realism and authenticity, which are both relevant factors in design, are not only achieved through physical resemblance (physical fidelity) but also require other fidelity factors [19]. Hence, future research in this field should be designed to also consider conceptual fidelity (scenarios and cases consistent with reality) and psychological fidelity (the ability to provoke emotional responses like reality) in the design of virtual simulations [19], factors that were not considered in this review.

Nevertheless, we identified 2 tools that had specifically used recordings of real people in the clinical situations being trained, which could have generated a greater feeling of immersion among students because of the increased physical, auditory, and visual fidelity of these tools. However, in the interactions with the simulation developed by Bearman et al [23], users had to respond from a pool of pre-established options, limiting the immersion experience because the participant was unable to develop their own communication skills in the way they would have to when facing real situations. In a tool developed by Courteille et al [28], although the user had been allowed to issue a natural language response, this had to be done in writing, which also reduced the degree of reality and spontaneity one would expect from a real conversation. Therefore, highly immersive technologies must be designed to overcome these ongoing technological challenges, such as how to integrate effective natural language processing systems and natural conversation flows into these tools [60] and how to best capture nonverbal communication [17,18]. For example, in this review, we only identified 2 applications that could detect gestures and/or emotions [29,32].

Of note, most of the tools we identified were based on autonomous learning and therefore represented promising applications with potential great benefits such as high accessibility levels, the possibility of repeating the experience multiple times, and cost reduction once running [16,17]. In this sense, technological advances that can integrate systems that provide feedback to participants—such as AI and machine learning (ML)—without the need for an instructor/teacher to mediate the learning stand out in particular [60]. For example, compared to a previous literature review [18], we found more tools in which the feedback was provided by the virtual system itself. However, as discussed, despite cataloging the existence of various patient simulation tools with interesting characteristics, we did not identify any that simultaneously integrated the use of a real person (a standardized patient) with the objective of increasing the environmental fidelity to allow the user to train through an oral conversation using natural language and using complex technology, such as AI and ML, with the ability to detect, encode, and respond to complex communication structures [60].

Finally, it is important to note that there were several limitations to this review. First, we only consulted 2 medical databases—MEDLINE/PubMed and the Web of Science. Despite being a health science–specific database and a multidisciplinary database, respectively, having replicated the search in more technological databases may have provided some additional studies for consideration. Therefore, it is possible we did not recover all the relevant records on virtual simulation tools to train communication skills in health care professionals registered in the academic literature. Second, there is still inadequate standardization in academic and scientific fields regarding the term “virtual simulation” [16,55,61]. Thus, different terms in the academic literature are all used to refer to the concept of virtual simulation including “serious games,” “virtual worlds,” “virtual patients,” and “virtual reality,” [55] which may have also caused us to miss certain relevant records.

In conclusion, this review identified and analyzed the 24 main virtual tools described in the academic literature that have been used to date to train communication skills in the context of health sciences. The high heterogeneity in terms of their characteristics means that tools based on AI and ML that contribute to training both students and practicing health professionals with as high a fidelity as possible to real life remain to be developed. Although many tools offer a degree of realism, few incorporate advanced features like AI-driven conversational flows or nonverbal cue detection, limiting the immersive experience. This highlights a need for further development to create more effective training environments. Addressing these gaps requires future innovations that integrate natural language processing and other advanced capabilities to enhance both the realism and educational value of virtual simulations.

## Supplementary material

10.2196/63082 Checklist 1 PRISMA (Preferred Reporting Items for Systematic Reviews and Meta-Analyses) checklist.

## Abbreviations

AI: artificial intelligence
MeSH: Medical Subject Headings
ML: machine learning
PRISMA: Preferred Reporting Items for Systematic Reviews and Meta-Analyses
